# Hepatitis B vaccination coverage among healthcare workers at national hospital in Tanzania: how much, who and why?

**DOI:** 10.1186/s12879-017-2893-8

**Published:** 2017-12-20

**Authors:** Dotto Aaron, Tumaini J. Nagu, John Rwegasha, Ewaldo Komba

**Affiliations:** 1 School of Medicine, Muhimbili University of Health and Allied Sciences, P.O. Box 65001, Dar es Salaam, Tanzania; 2 Muhimbili National Hospital, P.O. Box 65000, Dar es Salaam, Tanzania

**Keywords:** Vaccine, Hepatitis, HBV, Prevention, Liver cirrhosis, Hepatoma, SSA, Sub-Saharan Africa, Tanzania

## Abstract

**Background:**

Hepatitis B vaccination for healthcare workers (HCWs) is a key component of the WHO Hepatitis B Elimination Strategy 2016–2021. Data on current hepatitis B vaccine coverage among health care workers in Sub-Saharan Africa are scarce, but these data are vital for effective programming. We assessed the proportion of HCWs vaccinated for hepatitis B and the factors associated with adequate vaccination coverage at a national hospital in Tanzania.

**Methods:**

A descriptive cross-sectional study was conducted among consenting healthcare workers between 30th July and 30th September 2015. Vaccination histories were obtained through self-administered questionnaires. Means and proportions were used to summarize the data. Student’s t and chi-squared tests were used as appropriate. Logistic regression was used to determine the factors associated with vaccination.

**Results:**

A total of 348 HCWs were interviewed, of whom 198 (56.9%) had received at least one dose of hepatitis B vaccination, while only 117 (33.6%) were fully vaccinated. About half of the 81 HCWs with partial vaccination (49.4%) had missed their subsequent vaccination appointments. Among unvaccinated HCWs, 14 (9.3%) had either HBV infection or antibodies against HBV infection upon pre-vaccination screening. However, the remaining participants were not vaccinated and did not know their immune status against HBV.

Nearly all respondents (347, 99.3%) had heard about the hepatitis B viral vaccine. The following reasons for non-vaccination were given: 98 (65.3%) reported that they had not been offered the vaccine; 70 (46.7%) observed standard precautions to ensure infection prevention and 60 (41.3%) blamed a low level of awareness regarding the availability of the hepatitis B vaccine.

**Conclusion:**

The current vaccination coverage among practicing healthcare workers at Muhimbili National Hospital is low, despite a high level of awareness and the acceptance of the vaccine. Expedited and concerted efforts to scale vaccine uptake should include improved access to the vaccine, especially for newly recruited HCWs. The extension of the study to private healthcare settings and lower-level facilities would be useful.

## Background

Viral hepatitis infection was the seventh leading cause of global mortality and was responsible for 1.5 million deaths and 42 million disability-adjusted life years (DALYs) in 2013 [1]. Among the hepatitis viruses, Hepatitis B and C are responsible for more than 90% of the global burden of viral hepatitis [1]. It is estimated that about 240–257 million people have chronic HBV infections around the world [1]. The global prevalence of HBV infection is 1.3% and varies by geographical setting from as low as 0.2% in America to 3% in Africa [1]. More than two-thirds of hepatitis patients are in Sub-Saharan Africa and East Asia, where the prevalence is more than 8% [1–3]. The Hepatitis B vaccine has been available since 1981 and is considered a key strategy for preventing HBV infections.

HBV is endemic in East Africa, with an estimated HBsAg prevalence of 8% [1, 3]. In Tanzania, the HBV prevalence varies from one population to another, ranging between 8.8 and 11% among blood donors, [4–6] between 4 and 8% among pregnant women [7–10] and between 8 and 17% among HIV-infected individuals [11]. A study performed in Uganda showed that the sero-prevalence of HBV infection in the general population was 10% [12], and in Kenya, a recent national survey reported an HBV prevalence of 2.1%, with some regions recording a prevalence of 7.5% [13]. Earlier studies in Kenya reported an HBV prevalence between 8 and 10% [14, 15].

Healthcare workers (HCWs) are exposed to the constant risk of HBV infection due to their occupational contact with blood, blood products and other body fluids, as well as the risk of needle-stick injuries. In East Africa, the prevalence of HBV among HCWs is estimated to be between 7 and 8% [16, 17]. In developing countries, 40–65% of HBV infections among healthcare workers are attributable to percutaneous occupational exposure, whereas the corresponding risk in developed countries is as low as 10% [18]. A safe and effective HBV vaccine is available and recommended by the world Health Organization (WHO) for the primary prevention of HBV among all healthcare workers [19]. The introduction of the Hepatitis B vaccine, along with health education, reduced HBV prevalence from 10% to 1% among healthcare workers in India [19–21].

In Tanzania, the Hepatitis B vaccine is offered without charge to all public healthcare workers. However, only one in five healthcare workers were found to have protective immunity resulting from vaccination in a tertiary referral hospital in Tanzania [17]. In such a setting, it is important to assess the facilitators of and hurdles to HBV protection among healthcare workers. This study therefore seeks to determine the proportion of HCWs vaccinated against HBV, as well as to un-cover the factors associated with vaccination status. The findings of this study will guide policy and interventions in similar settings, particularly in Sub-Saharan Africa (SSA), as the global community works toward the elimination of HBV.

## Methods

### Design, patients and study procedures

A descriptive cross-sectional study was conducted at Muhimbili National Hospital (MNH), Dar es Salaam, from 30th July to 30th September 2015. The study included healthcare providers working in all departments, including trained nurses, medical attendants, and clinicians – both surgical and medical-related specialties and laboratory technicians. The investigator approached healthcare workers through the hospital administration and the heads of the departments. A self-administered questionnaire was used to obtain information from the participants. The questionnaires were collected from the healthcare workers upon completion by appointment with the participant. The sample size was calculated using the following formula for cross-sectional studies: N = {Z^2^P (1 – P)} /D^2^, where N = minimum sample size required; Z = standard normal deviation, which was set at 1.96; P = estimated proportion of healthcare workers vaccinated for HBV, which was unknown during the planning of this research and was estimated at 50%, and D = type 1 error, which we set at 0.05. The required sample size was 384.

### Definition of terms

#### HBV vaccination schedule

HBV vaccination is provided in the form of an intramuscular injection in three doses. The first dose is the baseline dose, and the second and third doses are provided one and 6 months after the first dose, respectively.

### Complete HBV vaccination

Healthcare workers who had received three HBV vaccine doses according to the HBV schedule were considered to have complete HBV vaccination.

### Incomplete but on schedule HBV vaccination

Healthcare workers who had received one or two HBV vaccine doses according to the schedule but were not due for their next dose were considered to have a vaccination level that was incomplete but on schedule.

### Incomplete HBV vaccination

Healthcare workers who had not received a full vaccination course but who were otherwise 1 month or more post-appointment for their scheduled dose were considered to have incomplete vaccination.

### Ineligible for HBV vaccination

Healthcare workers with HBV infection or immunity to HBV upon their vaccination screening visit were considered ineligible for HBV vaccination.

### Statistical analysis

The information requested via the questionnaire included vaccination status, knowledge about the transmission of and protection against HBV, and attitudes toward the Hepatitis B vaccine. The questionnaires were initially prepared in English, translated into Kiswahili and then back-translated in English to ensure that there was no loss of meaning. Both questionnaires were pre-tested for clarity and adjusted accordingly. The data were analyzed using Statistical Software for the Social Sciences (SPSS) Version 20. The categorical variables were summarized using proportions, while means were used to summarize the continuous variables. The variable of interest was complete or on-schedule vaccination as per the hepatitis B vaccination schedule. The differences between participants with complete/on-schedule vaccination and those with incomplete or no vaccination were tested using student’s t- and chi-squared tests as appropriate. Logistic regression was used to investigate the factors associated with complete and on-schedule vaccination. HCWs who were ineligible for HBV vaccination were removed from the comparative analyses.

### Ethics, consent and permissions

Ethical clearance was sought from the Muhimbili University of Health and Allied Sciences (MUHAS) review board. Permission to conduct the study was sought from the MNH administration. Informed written consent was obtained from participants before recruitment into the study. Confidentiality and privacy were ensured, and the information provided was stored securely, with access being limited to the investigators. No individual information or data that could lead to identity disclosure are published in this manuscript.

## Results

During the study, 348 healthcare workers (HCWs) were interviewed at the hospital, of whom 158 (45%) were males and 190 (54.5%) were females. Table 1 provides the demographic characteristics of the study participants. The mean age was 33 years, and half of the participants were 30 years old or younger. More than three-quarters of the participants had worked for 10 years or fewer. The nurse cadre was predominant in the study group, making up 124 (35.6%) of the participants. Specialist doctors were the least common, at 22 participants (6.3%). Details regarding these demographic characteristics are provided in Table 1.

**Table 1:** General characteristics of the Health Care Workers at Muhimbili National Hospital (*n* = 348)

Variable	Frequency (%)
Gender	
Male	158 (45.4)
Female	190 (54.6)
Age group (years)	
21–30	191 (54.9)
31–40	94 (27.0)
41–50	44 (12.6)
>50	19 (5.5)
Medical cadre	
Intern doctor	48 (13.8)
Registrar/resident	62 (17.8)
Specialist	22 (6.3)
Nurse	124 (35.6)
Hospital attendants	48 (13.8)
Laboratory technicians	44 (12.6)
Duration of employment (years)	
0–1	97 (27.9)
2–5	106 (30.5)
5–10	71 (20.4)
11–20	42 (12.1)
>20	32 (9.2)
Department	
Medical/pediatrics	96 (27.6)
Surgical and ^a^OBGY	81 (23.3)
Theater/Laboratory	76 (21.3)
^b^EMD/^c^ICU/Mortuary	49 (14.1)
^d^OPD	46 (13.2)

^a^OBGY = Obstetrics and Gynecology

^b^EMD = Emergency department

^c^ICU = intensive care unit

^d^OPD = Outpatient department

Table 2 shows the vaccination status of the interviewed HCWs. Partial or full vaccination for the Hepatitis B virus was reported by 198 (56.9%) interviewees. However, only 117 (33.6%) were fully vaccinated, with three doses of the vaccine. Among HCWs with partial vaccination, 41 (50.6) were not yet due for their next dose according to the vaccination schedule. The remaining half, some 40 individuals (49.4%) with partial vaccination, had missed their scheduled vaccination visit for 1 month or more. Among unvaccinated HCWs, 14 (9.3%) had either HBV infection or antibodies against HBV infection upon pre-vaccination screening.

**Table 2:** Hepatitis B Vaccination status among Health Care Workers at Muhimbili National Hospital (*n* = 348)

	*n*(%)
HB vaccination status	
Only one HB vaccine dose	30 (8.6)
Two HB vaccine doses	51 (14.7)
Full vaccination (3 doses)	117 (33.6)
Un-vaccinated	150 (43.1)
Vaccination status among HCW with Partial vaccination^a^	
Partial vaccination on schedule	41 (50.6)
Partial vaccination missed appointments	40 (49.4)

^a^Among those with partial vaccination

Table 3 provides an unadjusted comparison of vaccination coverage among the interviewed HCWs by department. Outpatient departments had the lowest proportion of vaccinated HCWs, at ten individuals (22.7%), while emergency departments, intensive care units (ICUs) and mortuary departments had the highest proportion of vaccinated individuals (76.6%) (Table 3). Healthcare workers (HCWs) who underwent partial or full-course vaccination had a mean employment duration 2.3 years, as compared to 2.5 years among those who were not vaccinated (*p* = 0.72).

**Table 3:** Comparison of vaccination status by department the participant worked at the time of interview (*n* = 334)^a^

	Number (%)		
Department^**^	Vaccinated *n* = 198	Unvaccinated *n* = 136	Total *n* = 334
Medical/Pediatrics	63 (67.0)	31 (33.0)	94
Surgical/^b^OBGY	48 (63.2)	28 (36.8)	76
Theater/laboratory	41 (56.2)	32 (43.8)	73
^c^EMD/^d^ICU/Mortuary	36 (76.6)	11 (23.4)	47
^e^OPD	10 (22.7)	34 (77.3)	44

^a^excluding HCW with HBV infection or HBV immunity at screening

^**^ *P* < 0.001

^b^OBGY = Obstetrics and Gynecology

^c^EMD = Emergency department

^d^ICU = intensive care unit

^e^OPD = Outpatient department

Regarding knowledge of the transmission and prevention of HBV, the majority of the respondents, 342 individuals (98.3%), had heard about hepatitis B viral infection prior to the interview. A total of 337 (96.8%) HCWs correctly identified their work as increasing the risk of acquiring HBV infection.

Unvaccinated health workers provided the following reasons for non-vaccination: they had not been offered a chance for hepatitis B vaccination (98 individuals, 65.3%), or they were very careful and observed standard precautions while at work (70 individuals, 46.7%) (Table 4). Others reported that there was not enough awareness concerning access to hepatitis B vaccination (62 individuals, 41.3%) (Table 4).

**Table 4:** Reasons for non-vaccination status among health care personnel at Muhimbili National Hospital (*n* = 150)

Reasons	Frequency (%)
I have never heard about the vaccine before	7 (4.7)
The vaccine is not available at my working place	44 (29.3)
I have not been offered a chance for HBV vaccination	98 (65.3)
I have no time for hepatitis B vaccination, very busy schedule	27 (18.0)
I am very careful, I observe standard precautions when I work	70 (46.7)
There is no enough education concerning HBV vaccination	62 (41.3)
I can’t afford the HBV vaccine	21 (14.0)
I was found infected on initial screening	8 (5.3)
I had HBsAb already on screening	10 (6.7)

Table 5 shows attitudes towards the hepatitis B vaccine. Among the respondents, 326 (93.7%) agreed that the Hepatitis B vaccine was effective in preventing HBV infection, while a few either disagreed (seven individuals, 2.0%) or were unsure about the effectiveness of the hepatitis B vaccine (15 individuals, 4.3%). Similarly, to a large extent, HCWs reported having trust in the vaccine (312 individuals, 89.7%), while a few (15 individuals, 4.3%) did not trust hepatitis B vaccine or had doubts about it (21 individuals, 6%). When we enquired into whether the hepatitis B vaccine should be compulsory among HCWs in Tanzania, 333 (96.8%) of the respondents agreed, while seven (2%) disagreed and four (1.1%) were undecided about mandatory hepatitis B vaccination for HCWs.

**Table 5:** Attitude towards hepatitis B vaccine as reported by health care workers at Muhimbili National Hospital (*n* = 348)

	Agree *n* (%)	Disagree *n* (%)	Undecided *n* (%)
Effective for the disease prevention	326 (93.7)	7(2.0)	15 (4.3)
I trust hepatitis B vaccine	312 (89.7)	15 (4.3)	21 (6.0)
The vaccine is not safe	17 (4.9)	225 (73.3)	76 (21.8)
Should be compulsory for all health care workers	333 (96.8)	7 (2.0)	4 (1.1)

Based on the multivariate logistic regression analysis, medical attendants (PR 6.6; 95% CI 2.0–21.7) and laboratory technicians (PR = 5.7; 95% CI 1.4–22.6) were more like likely to have incomplete or no vaccination compared to interns. Similarly, newly recruited HCWs were more likely to be associated with incomplete or non-vaccination status as compared to HCWs who had been working for more than 10 years (Table 6). It is worth noting that HCWs working in the outpatient department were significantly less likely to be vaccinated as compared to HCWs from medical (internal medicine and pediatrics) departments (PR 7; 95% CI 2.0–21.7). The detailed multivariate analysis results are provided in Table 6.

**Table 6:** Multivariate analysis of factors associated with incomplete or non-vaccination status among health care workers at Muhimbili National Hospital (*n* = 334)^a^

	PR^b^	95.0% C.I.		*P*
Male	1.23	0.717	2.11	0.453
Age (years)	1.433	0.877	2.34	0.151
Cadre				
Intern doctors	1			
Registered doctors/specialists	0.578	0.208	1.605	0.293
Nurses	1.972	0.81	4.797	0.135
Medical attendants	6.611	2.018	21.659	0.002
Laboratory technicians	5.707	1.442	22.583	0.013
Work duration (years)				
0 -- 1	9.501	2.629	34.329	0.001
2 -- 5	10.148	2.921	35.254	<0.0001
6 -- 10	2.792	1.05	7.423	0.04
>10	1			
Work department				
Medical/Pediatrics	1			0
Surgical/ ^c^OBGY	1.256	0.64	2.464	0.507
Theatre/Laboratory	0.776	0.285	2.108	0.618
^d^EMD/^e^ICU/Mortuary	0.472	0.2	1.114	0.087
^f^OPD	7.815	2.743	22.263	<0.0001

^a^excluding HCW with HBV infection or HBV immune at screening

^b^PR = Prevalence ratio

^c^OBGY = Obstetrics and gynecology

^d^EMD = Emergency department

^e^ICU = intensive care unit

^f^OPD = Outpatient department

## Discussion

In this study, we have observed that only one in three HCWs at the national referral hospital had undergone full hepatitis B vaccination, despite high levels of awareness and the near complete acceptance of the vaccine. These rates are lower than those in Europe 50–90% [22–29] and North America 63.4% [30]. Hepatitis B vaccine coverage is even lower in the neighboring countries of Kenya (12%) [31] and Uganda (5%) [32]. These low vaccination rates are worrisome because accidental exposure to blood and body fluids is common among healthcare workers [32–34] and has been associated with occupational hepatitis B infections. Efforts must be made to increase coverage.

Addressing incomplete vaccination (81 individuals, 23%) is also important, particularly among those who have already missed their scheduled visit and are thus potential defaulters on the vaccination program (40 individuals, 11.4%). These HCWs are potentially at risk for infection should they be exposed to HBV. A full-course hepatitis B vaccine (three doses) is safe and tolerable and offers 95–100% protection for adults [35, 36]. The first and second HBV vaccine doses provide less protection, specifically up to 85% protection against HBV infection [35]. A significant proportion of the HCWs who received two doses and even more of those who received only one dose of the vaccine are at risk of contracting hepatitis B infection should they be exposed. This message must clearly delivered to HCWs because incomplete vaccination is largely attributed to carelessness or forgetfulness [31]. There should be no misconceptions regarding HCWs having full protection with less than three doses without demonstrated evidence of immunity through antibody level assessment.

Improving hepatitis B vaccination coverage among healthcare workers at MNH requires addressing various bottlenecks. Based on previous studies, as well as the findings from our study, hepatitis B knowledge [14, 23], access to hepatitis B vaccination [31] and a lack of publicity are the major stumbling blocks to increased coverage [31, 38, 39]. Our study revealed that 47% of unvaccinated HCWs thought that observing infection control precautions and being careful would be enough to prevent HBV infection at their workplace. Healthcare personnel who view their susceptibility to HBV infection as high are more likely to be vaccinated than their counterparts who view their susceptibility to infection as low [23]. In other studies, those who wore gloves all or most of the time when they cared for patients or instruments were more likely to be vaccinated [37]. Appropriate knowledge about the transmission and prevention of HBV infection is vital in this regard. Two out of the three unvaccinated HCWs who responded reported that they had not been offered hepatitis B vaccination. Increased publicity for the vaccine would empower HCWs to demand vaccine and know how to access the vaccine. Reports show that publicity campaigns regarding the vaccination of HCWs yielded nearly complete protection rates among HCWs [38, 39]. Given the high acceptance rates regarding the vaccine among the HCWs in our study (97%) and those in Uganda (98%) [32], publicity is important in improving vaccine coverage. Therefore, a vaccination program, when properly planned and guided with policies such as mandatory vaccination among HCWs, could be successful [29, 40]. In European countries, policies differ; some countries enforce the mandatory vaccination of HCWs [41].

Multivariate analysis demonstrated that, laboratory technicians and medical attendants were six and seven times more likely to be unvaccinated/incomplete vaccination as compared to intern doctors. This finding is worrisome because the risk of occupational injury and exposure to HBV is higher among nurses and intern doctors [22, 42–44]. In other places such as India, vaccination for HBV was highest among interns and lowest among nursing attendants [45]. Our data, suggest that the majority of HCWs who worked at the outpatient department, were less likely to be vaccinated. Previous reports have shown that higher vaccination coverage has been associated with surgical and laboratory departments, where the risk is high [44]. It is therefore important that these departments be sensitized to and educated about the risks and advantages of vaccination. The locations for vaccine administration, the conflicting schedules for vaccine administration vs. duty stations, and the varying workloads among departments should be considered when designing and implementing HCW vaccination programs at a given health facility. Similarly, there was an association between the probability of non−/incomplete vaccination and increased employment duration. Education must particularly target recent employed health personnel to reduce risk of new hepatitis infections.

This study has shown the level of awareness of HBV infection among HCWs. Through first-hand information gathered via self-administered questionnaires, we were able to assess HCWs’ acceptance of the hepatitis B vaccine and address bottlenecks regarding increased uptake. Notwithstanding this important contribution, our study has certain weaknesses. Vaccination status was assessed through self-provided information, which is prone to recall bias. Given the fact that the participants were healthcare workers and recent national efforts to vaccinate healthcare workers in the country, we believe this bias to be very low. Vaccination does not always translate into immunity. Therefore, the use of a hospital database and coupling this study with an assessment of anti-HBsAb titres would have helped to inform policy change. Secondly, HCWs from all departments were approached through the administration, regardless of cadre and duration of employment. Purposeful selection would probably have increased the number specialist doctors included in the study.

## Conclusion

Hepatitis B vaccination coverage at this national hospital in Tanzania is currently low, despite good knowledge of and positive attitudes towards the vaccine. More effective programming, including publicity, as well as increasing access to the vaccination, could improve hepatitis B vaccine coverage. Feasibility studies regarding mandatory vaccination as a pre-requisite for employment should be conducted. Similar studies at private and lower-level health facilities are also warranted.

## Abbreviations

DALYs: Disability-adjusted life years
EMD: Emergency medicine department
HBsAb: Hepatitis B surface antibody
HBsAg: Hepatitis B surface antigen
HBV: Hepatitis B virus
HCW: Health care worker
ICU: Intensive care unit
IDU: Injection drug use
MNH: Muhimbili National Hospital
MUHAS: Muhimbili University of Health and Allied Sciences
OBGY: Obstetrics and gynecology
OPD: Outpatient department
PR: Prevalence ratio
SPSS: Statistical software for the social sciences
WHO: World Health Organization
